# Effect of Sustained Acoustic Medicine on Bruising Following A Bicycle Crash

**Published:** 2020

**Authors:** David O. Draper

**Affiliations:** Department of Exercise Sciences, BYU, USA.

## Abstract

In late September, 2020 a man was riding his mountain bike on a trail. Half-way into his ride his bike flipped and he fell off. His hip hit a rock. On examination 1 hour later he noticed a severe bruise on his hip. He immediately applied RICES for the next 2 days, except when he was sleeping. His on-off ratio for the RICES was 90-min “on”, 90 “min” off. Two days later he quit using RICES. Three days later he used sustained acoustic ultrasound (SAM) a small, portable ultrasound that can be used for up to 4hrs. Photos were taken after the 3^rd^ and 6^th^ treatment. There was lots of flesh colored tissue on the skin where the leg was treated with SAM.

## Case Report

**Fig1.**
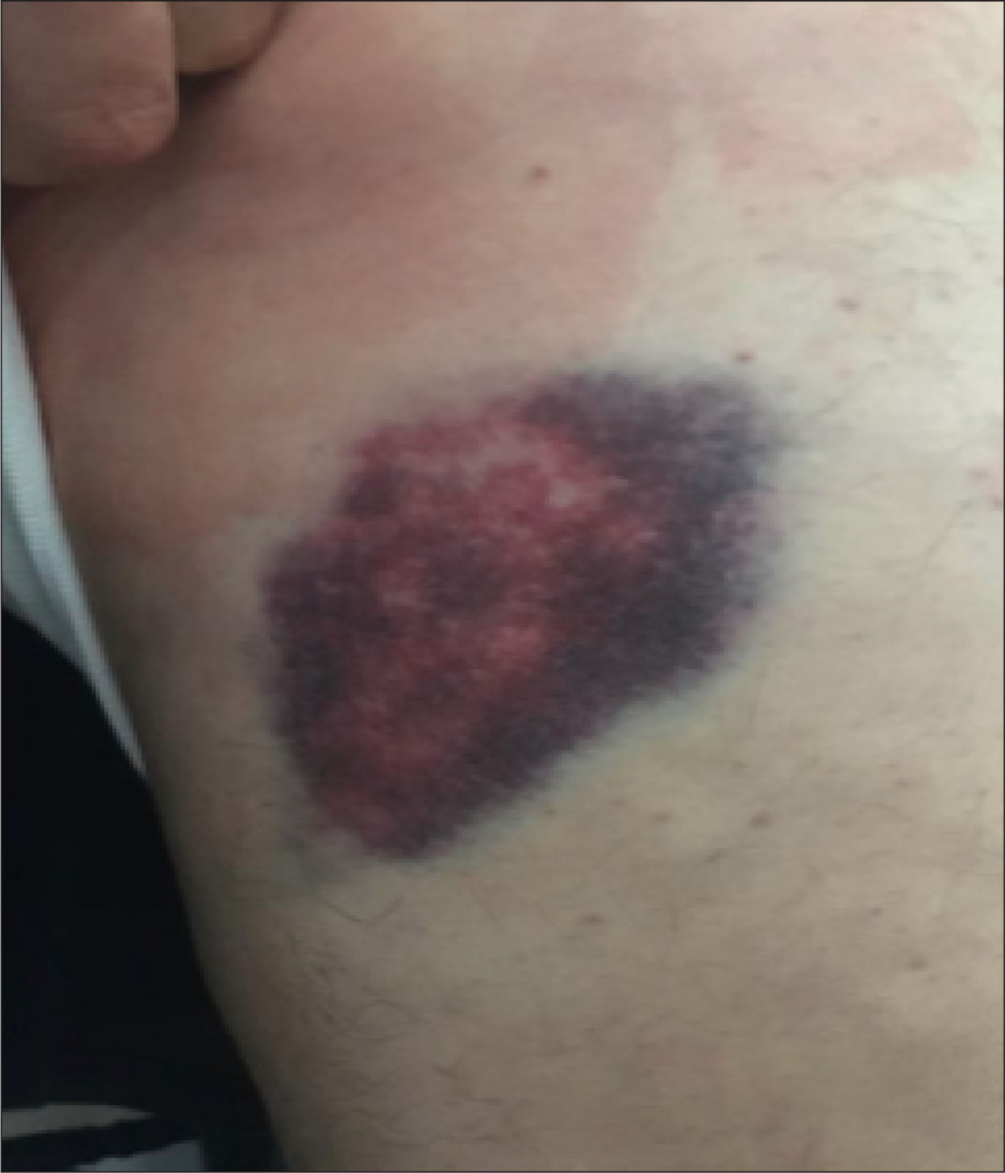
Bruise 48 hours after RICES

On September 28^th^, 2020 I was riding my E-bike on a mountain trail. I hit a slick spot, fell off my bike and my hip hit a rock. I was in a lot of pain, but continued my ride. When I returned home an hour later I rested my hip, applied an ice pack, elastic wrap, elevated the injury and stabilized it. (RICES). I applied ice for 1–2 hours at a time, followed by no ice for 1–2 hours. Nothing was applied at night. In the 1^st^ 48 hours I followed this for 6–7 times total. After this, the bruise was very dark (fig 1).

A few days later I implemented sustained acoustic medicine (SAM) at the following parameters:

- intensity (0.132W/cm^2^)
- duration (2 hours)
- frequency (3Mhz)
- joules (>9,000 per TX)
- total treatments (3 on Sat, 3 on Sun)

**Fig2.**
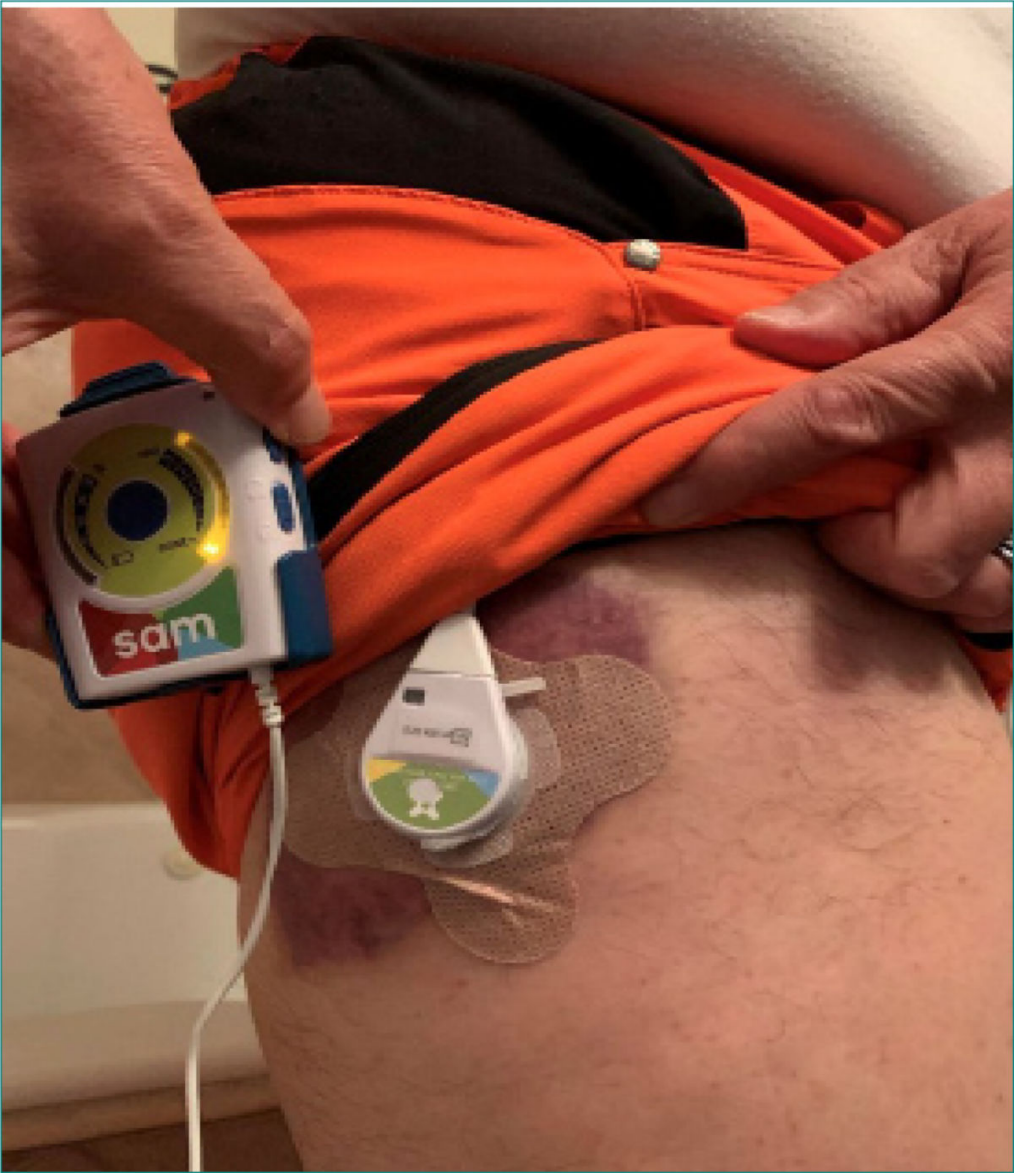
SAM used 3 times SAT for 2 hours

Figure 2 shows the application of SAM to the area.

**Fig3.**
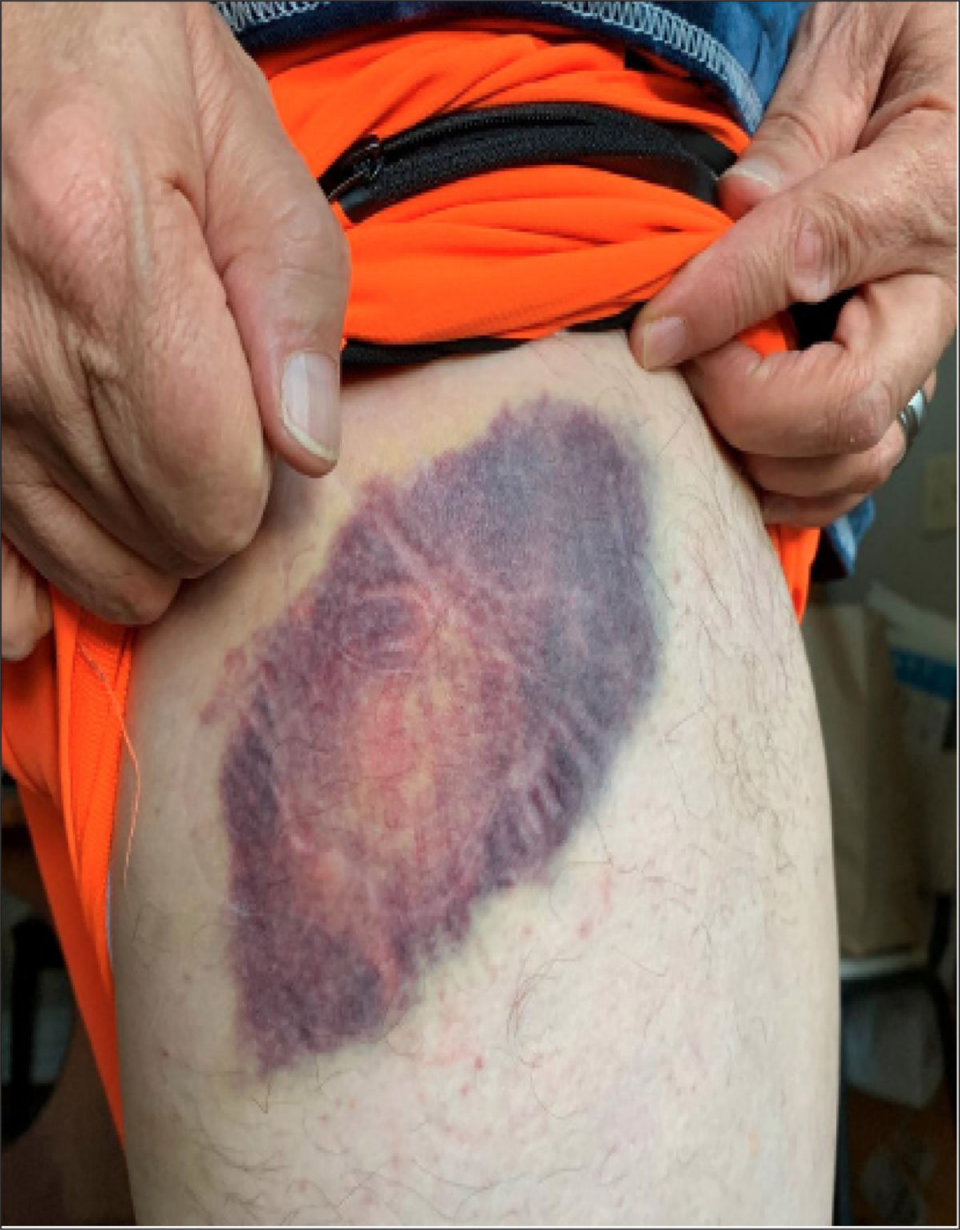
Bruise after 3 applications of SAM

Figure 3 shows the bruise after the 3 Saturday treatments.

**Fig4:**
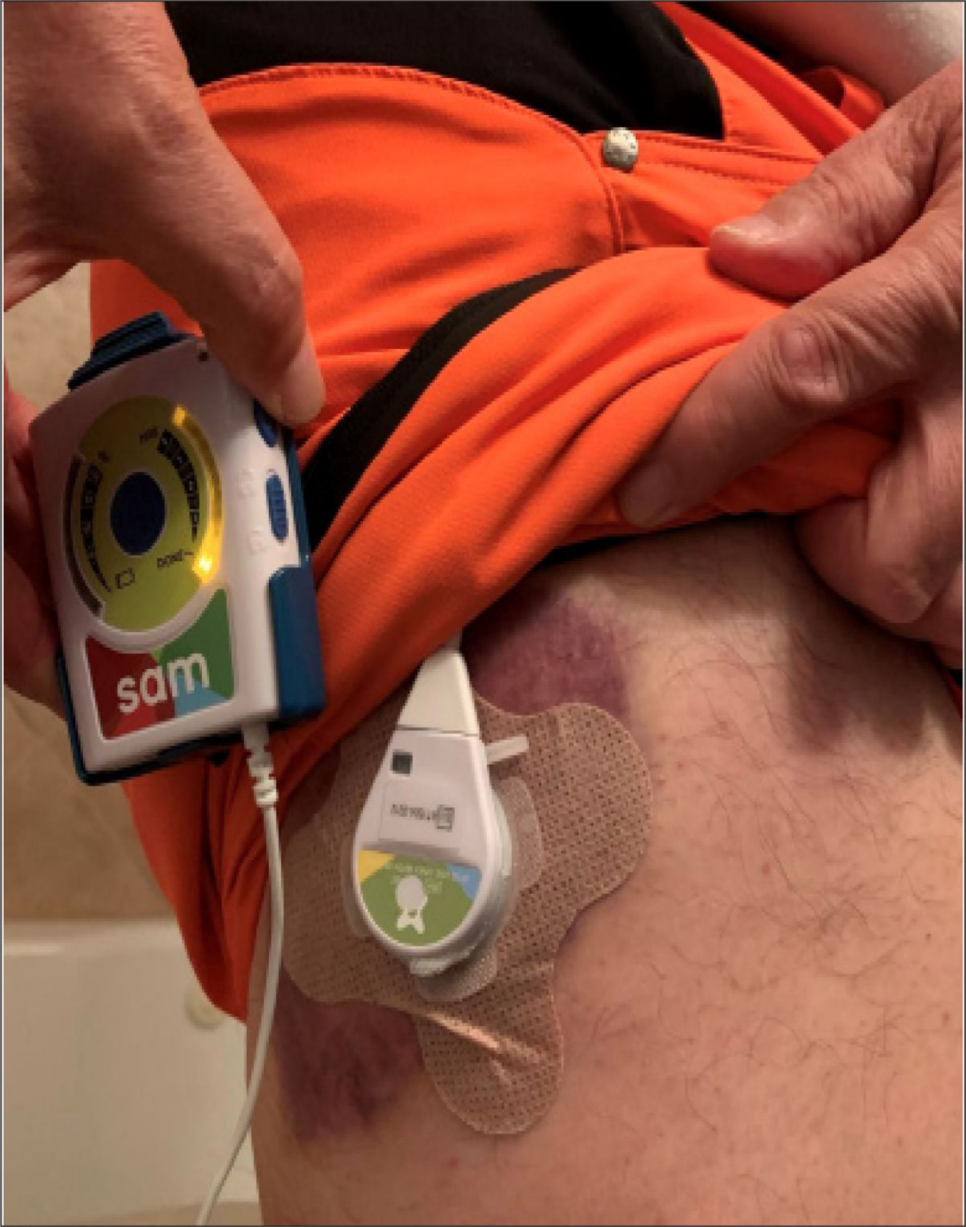
3 applications of SAM on Sunday

Figure 4 shows another application of SAM.

**Fig5.**
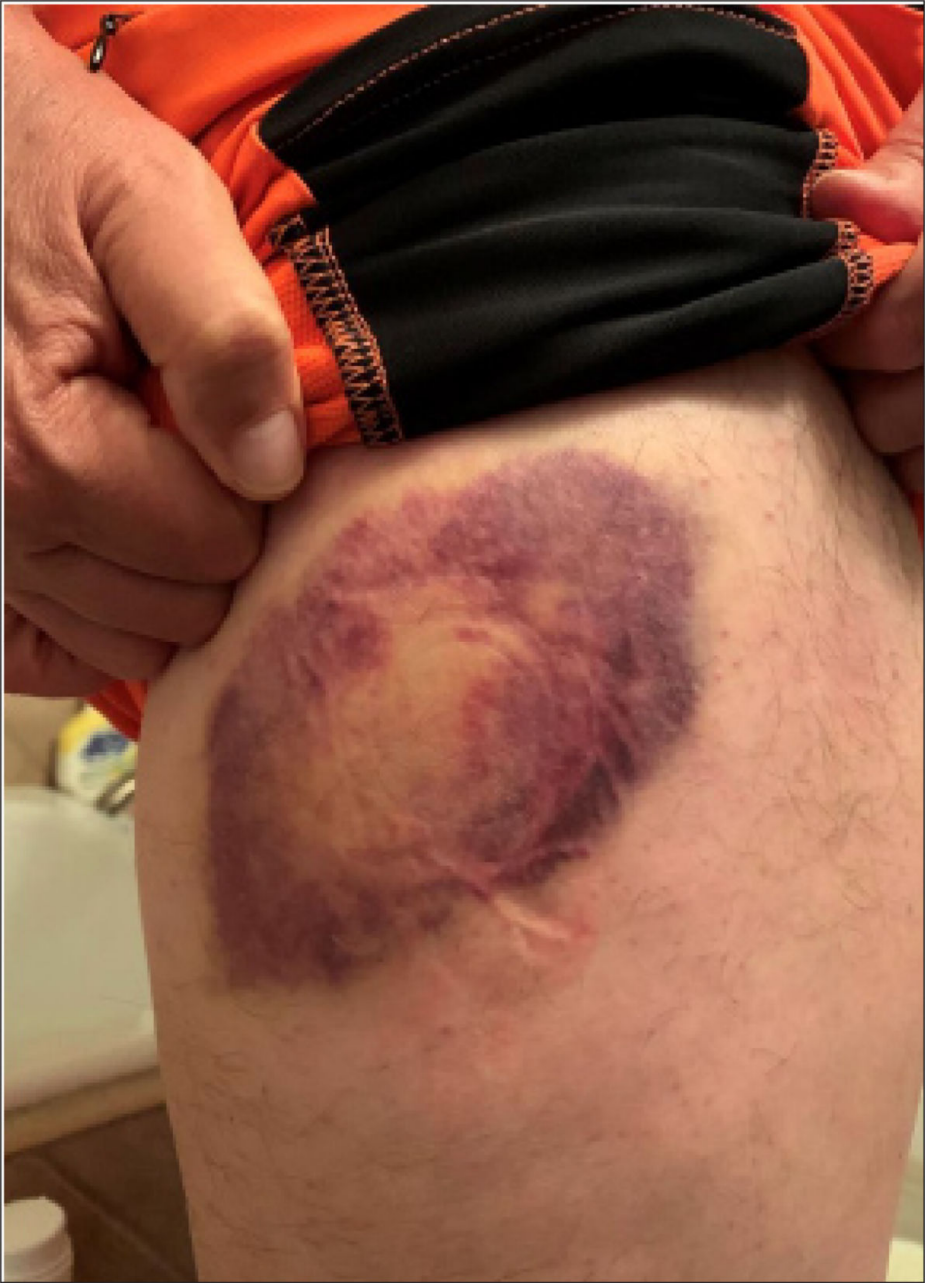
Bruise after 3 applications of SAM on Sunday (6 total applications)

Figure 5 shows the bruise bruise after the 3 treatments on Sunday. Note that where the 6cm^2^ crystal was applied, the bruise became lighter (Figs 2,3,4,5).

I was in a lot of pain except when RICES and SAM were applied. Ideally RICES should be applied within the 1^st^ 10 minutes after the injury to prevent excessive pain and swelling. For decades RICES has played a big role in treating acute injury.^1^ ***R*** stands for rest. I rested the injury by not biking or hiking for a few days. ***I*** stands for ice. Typically it is applied in a crushed form in a plastic bag. Even though many medical professionals disagree, the ice bag is applied directly on the skin. This will not cause frostbite unless a chemical cold pack is applied.^2,3,4^ ***C*** stands for compression and is applied with an elastic wrap. It should not be applied at maximum compression, but at 75% of this.^5^ ***E*** stands for elevation. Ideally the elevated structure should be above the heart. This can be applied by lying supine, with the leg rested on a pillow.^1^ **S** Stands for stabilization and can be applied by taping the affected part, or by putting a brace on it.^1^

SAM was created 15 years ago by George Lewis, PhD, while he was a doctoral student at Cornell. Today SAM is used by many professional sports teams in the U.S including: football, basketball, baseball, soccer, and ice hockey. SAM is classified as low-intensity therapeutic ultrasound (LITUS). It is different than typical thermal ultrasound in the following ways:

**Table:** 

TRADITIONAL THERAPEUTIC ULTRASOUND	SAM

Not portable	Portable
Delivers .01–2.5 W/cm^2^	Delivers 0.132 W/cm
Uses wall outlet	Uses batteries
1 and 3 Mhz frequency	only 3 Mhz frequency
Continuous and pulsed modes	Only continuous mode
Deep, medium and shallow depths	Medium and shallow depths
Needs clinician to deliver it	Self applied then works on its own
Energy: up to 5000 joules	Energy: up to 18,700 joules

As shown by the figures, SAM is not only effective in decreasing pain, healing of soft tissue and bruises.^6,7^
